# Spontaneous Ventilation Video-Assisted Thoracoscopic Surgery for Non-small-cell Lung Cancer Patients With Poor Lung Function: Short- and Long-Term Outcomes

**DOI:** 10.3389/fsurg.2022.800082

**Published:** 2022-03-02

**Authors:** Runchen Wang, Qixia Wang, Shunjun Jiang, Chao Chen, Jianqi Zheng, Hui Liu, Xueqing Liang, Zhuxing Chen, Haixuan Wang, Zhuoxuan Guo, Wenhua Liang, Jianxing He, Hengrui Liang, Wei Wang

**Affiliations:** ^1^State Key Laboratory of Respiratory Disease, Department of Thoracic Surgery and Oncology, National Clinical Research Center for Respiratory Disease, Guangzhou Institute of Respiratory Health, The First Affiliated Hospital of Guangzhou Medical University, Guangzhou, China; ^2^Nanshan School, Guangzhou Medical University, Guangzhou, China; ^3^Department of Anesthesia, The First Affiliated Hospital of Guangzhou Medical University, Guangzhou, China; ^4^School of Public Health, Southern Medical University, Guangzhou, China

**Keywords:** spontaneous ventilation video-assisted thoracoscopic surgery, poor lung function, non-small lung cancer, NSCLC, NIVATS

## Abstract

**Objective:**

The goal of this study was to explore the feasibility and safety of spontaneous ventilation video-assisted thoracoscopic surgery (SV-VATS) for non-small-cell lung cancer (NSCLC) patients with poor lung function.

**Methods:**

NSCLC patients with poor lung function who underwent SV-VATS or mechanical ventilation VATS (MV-VATS) from 2011 to 2018 were analyzed. 1:2 Propensity score matching (PSM) was applied, and the short- and long-term outcomes between the SV-VATS group and the MV-VATS group were compared.

**Results:**

Anesthesia time (226.18 ± 64.89 min vs. 248.27 ± 76.07 min; *P* = 0.03), operative time (140.85 ± 76.07 min vs. 163.12 ± 69.37 min; *P* = 0.01), days of postoperative hospitalization (7.29 ± 3.35 days vs. 8.40 ± 7.89 days; *P* = 0.04), and days of chest tube use (4.15 ± 2.89 days vs. 5.15 ± 3.54 days; *P* = 0.01), the number of N1 station lymph node dissection (2.94 ± 3.24 vs. 4.34 ± 4.15; *P* = 0.005) and systemic immune-inflammation index (3855.43 ± 3618.61 vs. 2908.11 ± 2933.89; *P* = 0.04) were lower in SV-VATS group. Overall survival and disease-free survival were not significantly different between the two groups (OS: HR 0.66, 95% CI: 0.41–1.07, *P* = 0.09; DFS: HR 0.78, 95% CI: 0.42–1.45, *P* = 0.43).

**Conclusions:**

Comparable short-term and long-term outcomes indicated that SV-VATS is a feasible and safe method and might be an alternative to MV-VATS when managing NSCLC patients with poor lung function.

## Introduction

Surgery is the primary approach to the treatment for the early stage of non-small cell lung cancer (NSCLC) (1). Traditional thoracotomy is controversial due to its severe complications and poor prognosis. Video-assisted thoracic surgery (VATS) has been widely used because it is minimally invasive and associated with less postoperative complications, shorter operations time and hospital stay compared with open thoracotomy (2–4).

General anesthesia with double-lumen intubation induced with mechanical ventilation during video-assisted thoracoscopic surgery (MV-VATS) has been widely recognized as a secure and effective thoracic anesthesia approach and is now considered a standard treatment option (5, 6). However, some adverse effects of the MV-VATS, including intubation-related airway trauma, impaired cardiac performance, and residual neuromuscular blockade, may pose uncertain risks for the surgery and affect the prognosis of NSCLC patients (7–10).

Spontaneous ventilation video-assisted thoracoscopic surgery (SV-VATS) has developed rapidly recently and achieved promising results (11). According to the reports, SV-VATS can reduce the perioperative adverse effects of tracheal intubation and general anesthesia in thoracic surgery (12–14) and provide more comfortable surgical experience for NSCLC patients. Therefore, SV-VATS has become a viable alternative to various thoracic surgeries (15–17).

Numerous studies have proved the feasibility and safety of SV-VATS in treating NSCLC (12, 13, 18–20). However, since lung cancer resection in patients with poor pulmonary function is considered risky and controversial (21–24), the safety and efficacy of SV-VATS in those patients remain unclear. This study aims to compare short- and long-term outcomes between the SV-VATS and the MV-VATS in treating patients with poor lung function.

## Materials and Methods

### Patients Inclusion

This study was reported under STROCSS 2019 guidelines (25). From January 2011 to July 2018, 5,857 NSCLC patients undergoing primary lung cancer resection at the First Affiliated Hospital of Guangzhou Medical University were consecutively identified and retrospectively collected through electronic records.

A total of 1,172 patients with poor lung function undergoing VATS were initially selected. According to the prior research (18, 22, 26–30) and following the recent GOLD guidelines (31), we define poor lung function as post-bronchodilator FEV1/FVC <70%.

The Institutional Review Board of the hospital reviewed the study protocol and methods, IRB report ID: 2018-57. Informed consent was obtained from every patient. Patients undergoing SV-VATS and MV-VATS were set as experimental and control groups, respectively. Patients were included based on the following criteria: (i) diagnosed with lung cancer based on pathological outcome; (ii) tumor size ≤ 5 cm; (iii) American Society of Anesthesiologists status class ≤ 3; (iv) Eastern Cooperative Oncology Group score ≤ 1; (v) patients underwent segmentectomy or lobectomy; (vi) no serious arrhythmia such as atrial fibrillation and frequent premature beat; and (vii) no cardiac insufficiency. Patients were excluded based on the following criteria: (i) a history of infection leading to massive pleural effusion, such as tuberculosis and pneumonia; (ii) a history of thoracic surgery. Patients and attending practitioners decided whether to receive SV-VATS or MV-VATS. Two independent radiologists performed enhanced CT scans for all included patients.

### Preoperative Preparation

Electrocardiograms, heart rate, respiratory rate, pulse oxygen saturation (SpO_2_), blood pressure, and bispectral index (BIS) were routinely monitored for patients after entering the operating room. Arterial catheters were routinely used to monitor the vital signs of the patient during the process of VATS surgery, such as blood gas analysis and the fluctuations in blood pressure. Central vein puncture were performed when necessary, so as to rescue the patient better and faster in unexpected circumstances during the operation. Atropine (0.01 mg/kg) was injected intravenously before performing anesthesia to prevent excessive airway secretions.

### SV-VATS Group

The combination of local nerve block and intravenous anesthesia was employed for SV-VATS. The patient was anesthetized as follows: (i) laryngeal mask airway (Royal Fornia Medical Equipment, Guangdong, China.); (ii) intravenous anesthesia; (iii) thoracic paravertebral block (7th and 8th thoracic vertebra); (iv) visceral pleural surface anesthesia (the visceral pleural surface was anesthetized with 5 mL of 2% lidocaine); (v) operated side thoracic vagus nerve block (under direct thoracoscopic view, 2 mL of 0.25% bupivacaine was infiltrated near the vagus nerve at the level of inferior trachea for the right side of procedure and the level of aortopulmonary window for the left side of procedure). Epidural block and other nerve block methods would be used when necessary. Anesthesia was induced with target-controlled infusion (TCI) of sufentanil 0.1–0.2 mg/kg and propofol 2.0–4.0 mg/mL. No muscle relaxant was used during the procedure. Sedatives such as propofol were used when BIS was greater than 60. A laryngeal mask airway would be inserted for patients if BIS dropped below 60. Anesthesia was maintained with TCI of propofol (target plasma concentration 0.5–1.0 mg/mL), inhaled sevoflurane (± 0.8–1.5-fold the minimum alveolar concentration), dexmedetomidine 0.05–0.10 μg/kg/min, remifentanil 0.05–0.15 μg/kg/min. To maintain BIS at 40–60 during operation, dexmedetomidine 0.5–1.0 mg/kg/h was added. To facilitate spontaneous ventilation, a laryngeal mask airway connected to the anesthesia machine was used. The third-generation double-tube LMA was used for ventilation management. If spontaneous ventilation cannot be used in the SV-VATS group, manual ventilation or simultaneous intermittent mandatory ventilation (SIMV) mode would be used to assist ventilation during anesthesia induction.

### MV-VATS Group

TCI of intravenous sufentanil (0.3–0.6 mg/kg), propofol (target plasma concentration 2–3 mg/mL), and cisatracurium (0.2 mg/kg) were administrated for anesthetic induction in the MV-VATS group. A double-lumen endotracheal tube was intubated via a visual laryngoscope 3 min after anesthetic induction. One-lung ventilation (OLV) under intermittent positive pressure ventilation model was applied for anesthesia maintenance period. The parameters of intermittent positive pressure ventilation mode were set as follows: the fraction of inspired oxygen (FiO_2_), tidal volume, 4–6 mL/kg; respiratory rate, 12–18 times/min; oxygen flow, 4–5 L/min; TCI of propofol (1.5–4 mg/mL), remifentanil (0.03–0.08 mg/kg/min), dexmedetomidine (0.5–1.0 mg/ kg/h), and cis-atracurium (0.05 mg/kg) were administered. BIS was maintained between 45 and 60 during operation. Dexmedetomidine was stopped directly after pleural cavity closure, while propofol and remifentanil were stopped at the end of operation. No inhaled anesthetic was used during the procedure.

### Surgical Process

The same surgical procedure was operated in the MV-VATS and the SV-VATS groups. Stryker 1288 HD 3-Chip Camera System (Stryker, Kalamazoo, MI, USA) was adopted as video-assisted thoracoscopic operations, and the endoscopic instruments were designed ourselves. Patients were placed in a lateral decubitus position with upper arms extended and fixed on the hand support.

According to the actual situation, surgery was carried out using a one-port or two-port approach for VATS. The thoracoscope was inserted at the 7th or 8th intercostal space on the anterior axillary line, which carried a soft incision protector to safeguard the skin, subcutaneous tissue, rib, and pleura.

Types of surgery included lobectomy and segmentectomy. N1 and N2 station lymph nodes were dissected routinely for all patients.

### Data Collection and Statistical Analyses

All data were extracted by two blinded, experienced abstractors, and a third abstractor judged conflicts.

This study integrated patient demographics, preoperative laboratory examinations, anesthetic, surgical information, postoperative recovery information from electronic medical records, and survival information via telephone or interviews. The systemic immune-inflammation index (SII), neutrophil-to-lymphocyte (NLR) ratio, and platelet-to-lymphocyte (PLR) ratio were calculated before and after surgery. SII was calculated as follows using formula: SII = platelet count ^*^ neutrophil count/lymphocyte count. Propensity score match (PSM) was adopted to reduce treatment selection biases. Patients who were lost to follow-up during the 90-day period were excluded from PSM. Factors including age, BMI status, smoking status, gender, pulmonary function, histological analysis, American Society of Anesthesiologists (ASA) status class, tumor position, T stage, N stage, M stage, and surgical technique were considered in PSM. Patients were matched and included in the experimental and control groups as a ratio of 1:2. The matching was performed using nearest neighbor method. Confounding variables were considered comparable when the standardized mean difference was below 0.10 and the *p*-value was greater than 0.05. Descriptive data were presented as means plus standard deviation, and categorical data were presented as percentages. The normality was examined using Shapiro-Wilk test. Student *t*-test or Wilcoxon test was performed for the comparison of inter-group continuous variables. Categorical variables were compared with Chi-squared test or Fisher's exact test. Kaplan-Meier was employed to estimate the overall survival (OS) and disease-free survival (DFS). A log-rank test was used to calculate *p*-values. All tests were 2-sided, and a *p* < 0.05 was considered statistically significant.

The follow-up strategy followed the NCCN guidelines (32). Specifically, physical examination and non-enhanced chest CT examinations were performed every 6 months for 3 years after surgery and annually after that and included a routine history. Follow-up phone calls were made until August 20th, 2021, to confirm survival status of patients.

Statistical analysis was performed using R 4.0.5 (The R Core Team, R Foundation for Statistical Computing, Vienna, Austria) running on R Studio 1.4.1106 (R Studio Team, R Studio Inc. Boston, MA, USA).

## Results

### Patient Baseline Characteristics and Demographics Data

**Figure 1:**
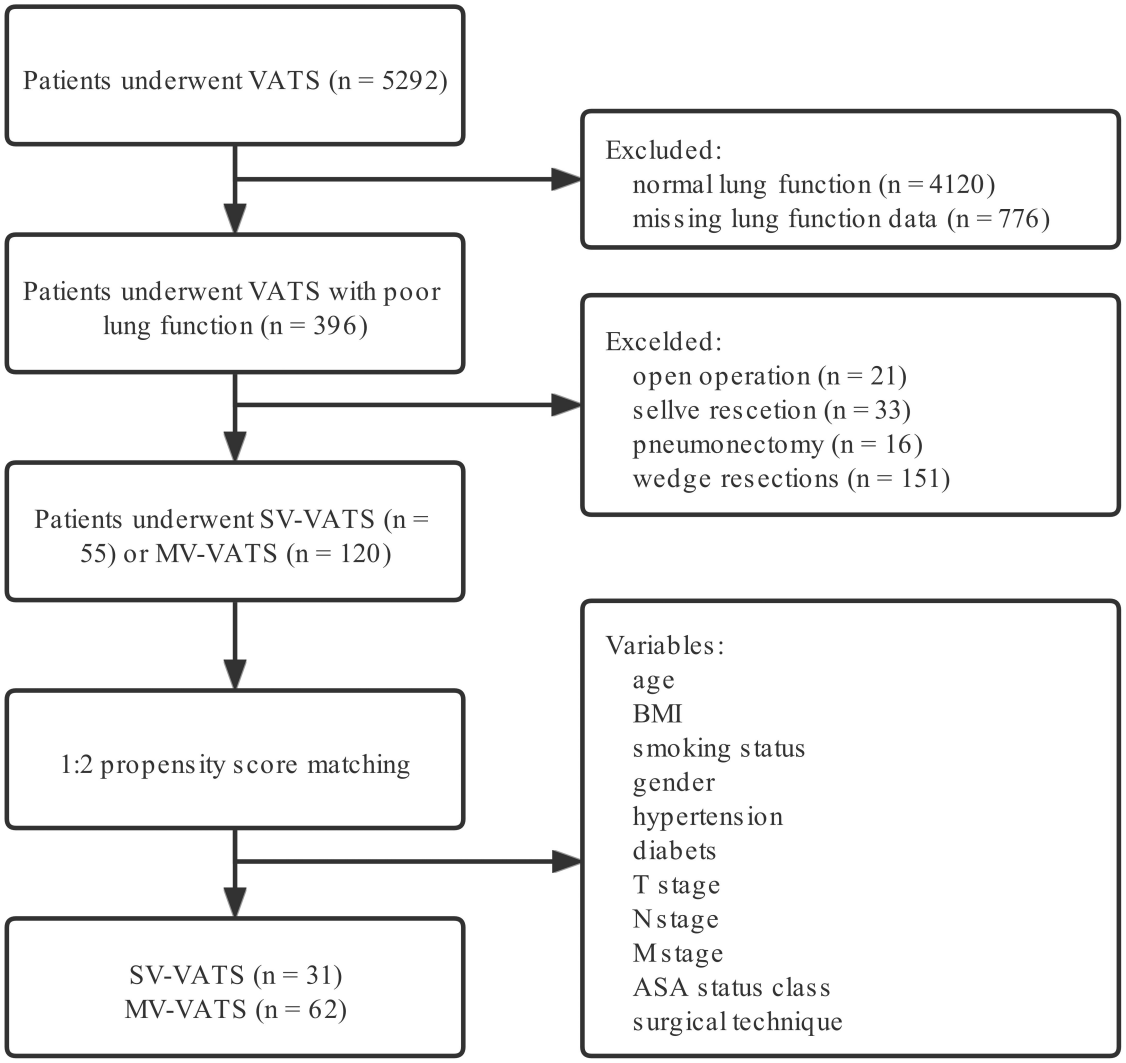
Flow chart of patient inclusion. NSCLC, non-small-cell lung cancer; SV-VATS, spontaneous ventilation video-assisted thoracoscopic surgery; MV-VATS, mechanical ventilation; BMI, body mass index; T, tumor; N, node; M, metastasis; ASA, american society of anesthesiologists.

The flow diagram of patient recruitment was displayed in Figure 1. Overall, 5,292 NSCLC patients underwent SV-VATS or MV-VATS were included in the study. Among the enrolled patients, 396 patients were presented with poor lung function.

After performing a 1:2 PSM, 24 patients in the SV-VATS group failed to match with control group and were excluded in the study (Supplementary Table 3). Finally, 31 and 62 patients were obtained from the SV-VATS and MV-VATS group, respectively. In the SV-VATS group, 2 patients underwent segmentectomy, while 29 patients underwent lobectomy. In the MV-VATS group, 7 patients underwent segmentectomy, while 55 patients underwent lobectomy.

FEV1/FVC (63 ± 7% vs. 61 ± 9%, *p* = 0.70) was similar between the two groups. Baseline demographic and clinical variables were well-balanced between the two groups (Table 1). The distribution of patient characteristics and propensity scores were shown in Supplementary Figures S1, S2.

**Table 1:** Distribution of preoperative confounders among patients with worse lung function (defined as FEV1/FVC <70%) in the SV-VATS and MV-VATS groups.

	**Before PSM**			**After PSM**		
	**SV-VATS/SD**	**MV-VATS/SD**	***P*-value**	**SV-VATS/SD**	**MV-VATS/SD**	***P*-value**
Age (years)			0.72			0.41
<60	9/5.90	26/9.08		8/4.95	10/7.45	
60–75	37/4.16	75/4.32		22/4.54	43/4.43	
> 75	9/2.06	19/4.26		1/0	9/1.47	
BMI			0.11			0.72
<18.5	3/0.56	13/0.76		1/0.22	5/0.22	
18.5–25	47/1.91	85/1.75		25/1.56	47/1.77	
> 25	5/1.08	22/1.81		5/0.97	10/0.10	
Smoking status			0.97			0.82
Never smoking	30/0.55	65/0.54		19/0.61	34/0.55	
Quit smoking	8/0.14	19/0.16		3/0.10	8/0.13	
Smoking	17/0.31	36/0.30		9/0.29	20/0.32	
Gender			0.44			0.73
Male	45/0.82	92/0.77		24/0.77	46/0.64	
Female	10/0.18	28/0.23		7/0.23	16/0.22	
Hypertension			0.90			0.68
Yes	17/0.31	36/0.30		4/0.13	10/0.16	
No	38/0.69	84/0.70		27/0.87	52/0.84	
Diabetes			0.24			1.00
Yes	0/0	3/0.03		0/0	0/0	
No	55/1	117/0.97		31/1	62/1	
T stage			0.83			0.98
1	35/0.63	77/0.64		2/0.06	39/0.63	
2	16/0.29	38/0.32		17/0.55	20/0.32	
3	2/0.04	2/0.02		11/0.36	1/0.02	
4	2/0.04	3/0.03		1/0.03	2/0.03	
N stage			0.30			0.73
0	44/0.80	81/0.67		22/0.71	43/0.69	
1	2/0.04	8/0.07		1/0.03	5/0.08	
2	8/0.14	30/0.25		7/0.23	13/0.21	
3	1/0.02	1/0.01		1/0.03	1/0.02	
M stage			0.72			0.58
0	51/0.93	113/0.94		28/0.90	58/0.91	
1	4/0.07	7/0.06		3/0.10	4/0.09	
ASA status class			0.38			0.15
I	1/0.02	3/0.03		0/0	1/0.02	
II	53/0.96	109/0.91		31/1	57/0.92	
III	1/0.02	8/0.06		0/0	4/0.06	
Surgical technique			0.39			0.27
Segmentectomy	9/0.16	14/0.12		2/0.06	7/0.11	
Lobectomy	46/0.84	106/0.88		29/0.94	55/0.89	

*MV-VATS, mechanical ventilation video-assisted thoracoscopic surgery; SV-VATS, spontaneous ventilation video-assisted thoracoscopic surgery; BMI, body mass index; T, tumor; N, node; M, metastasis; ASA, american society of anesthesiologists*.

### Intraoperative Outcomes Between SV-VATS and MV-VATS

Intraoperative bleeding volume (92.10 ± 175.40 mL vs. 158.40 ± 262.38 mL; *P* = 0.04) was less in the SV-VATS group than MV-VATS group. No significant difference was found in anesthesia time (241.84 ± 56.99 min vs. 258.47 ± 78.13 min; *P* = 0.66) and operative time (151.10 ± 46.80 min vs. 169.35 ± 76.63 min; *P* = 0.15) between SV-VATS and MV-VATS group.

During lymph dissection, no significant difference was found in number of N1 lymph node dissection (2.74 ± 2.99 vs. 4.87 ± 4.28; *P* = 0.06), group number of N1 station lymph node dissection (1.03 ± 0.98 vs. 1.21 ± 1.03; *P* = 0.86), number of N2 station lymph node dissection (9.97 ± 7.60 vs. 11.19 ± 8.38; *P* = 0.63) and group number of N2 station lymph node dissection (2.45 ± 1.34 vs. 2.65 ± 1.34; *P* = 0.74) between SV-VATS and MV-VATS group. All surgical outcomes were summarized in Table 2.

**Table 2:** Perioperative outcome after 1:2 propensity score matching.

	**SV-VATS**		**MV-VATS**		***P*-value**
	**Mean**	**SD**	**Mean**	**SD**	
Days of postoperative hospitalization (days)	7.74	5.41	9.97	7.95	0.20
Anesthesia time (min)	241.84	56.99	258.47	78.13	0.66
Operative time (min)	151.10	46.80	169.35	72.63	0.15
Intraoperative bleeding volume (ml)	92.10	175.40	158.40	262.38	0.04
Days of chest tube use (days)	4.10	2.29	4.87	2.96	0.44
N1 lymph node dissection					
Number	2.74	2.99	4.87	4.28	0.06
Group number	1.03	0.98	1.23	1.03	0.86
N2 lymph node dissection					
Number	9.97	7.60	11.19	8.38	0.63
Group number	2.45	1.34	2.65	1.34	0.74
SII before surgery	476.64	483.99	499.56	317.16	0.12
SII after surgery	3566.10	3239.61	4447.30	4355.61	0.87
NLR before surgery	2.50	1.21	2.24	0.94	0.44
NLR after surgery	18.72	14.98	21.15	16.54	0.02
PLR before surgery	109.46	84.64	119.80	58.62	0.10
PLR after surgery	317.88	198.63	415.46	322.35	0.04

*MV-VATS, mechanical ventilation video-assisted thoracoscopic surgery; SV-VATS, spontaneous ventilation video-assisted thoracoscopic surgery; N, node; SII, systemic immune-inflammation index; NLR, neutrophil-to-lymphocyte; PLR, platelet-to-lymphocyte*.

### Comparison of Short-Term Outcomes

Both of the days of chest tube use (4.10 ± 2.29 days vs. 4.87 ± 2.96 days; *P* = 0.44) and days of postoperative hospitalization (7.74 ± 5.41 days vs. 9.97 ± 7.95 days; *P* = 0.20) were comparable in two groups.

There was no statistically significant difference in preoperative inflammatory indicators between the SV-VATS and MV-VATS group (SII: 476.64 ± 483.99 vs. 499.56 ± 317.16; *P* = 0.12. NLR: 2.50 ± 1.21 vs. 2.24 ± 0.94; *P* = 0.44. PLR: 109.46 ± 84.64 vs. 119.80 ± 58.62; *P* = 0.10). After surgery, no statistical difference existed in SII (3556.10 ± 3239.61 vs. 4447.30 ± 4355.61; *P* = 0.87). However, NLR (18.72 ± 14.98 vs. 21.15 ± 16.54; *P* = 0.2) and PLR (317.88 ± 198.63 vs. 415.46 ± 322.35; *P* = 0.94) after surgery was significantly lower in SV-VATS group than MV-VATS group. The incidence of complications (16 vs. 24%, *P* = 0.37) was comparable in the two groups. The details of postoperative outcomes were summarized in Tables 2, 3.

**Table 3:** Perioperative complications after 1:2 propensity score matching.

**Complications**	**SV-VATS**		**MV-VATS**		
	**(*n* = 5)**	**Proportion (%)**	**(*n* = 15)**	**Proportion (%)**	***P***
Pneumothorax	4	12.90	11	17.74	0.55
Fever	1	3.23	1	1.61	0.61
Arrhythmia	0	0	2	3.23	0.31
Anemia	0	0	1	1.61	0.48

*MV-VATS, mechanical ventilation video-assisted thoracoscopic surgery; SV-VATS, spontaneous ventilation video-assisted thoracoscopic surgery*.

### Comparison of Long-Term Outcomes

**Figure 2:**
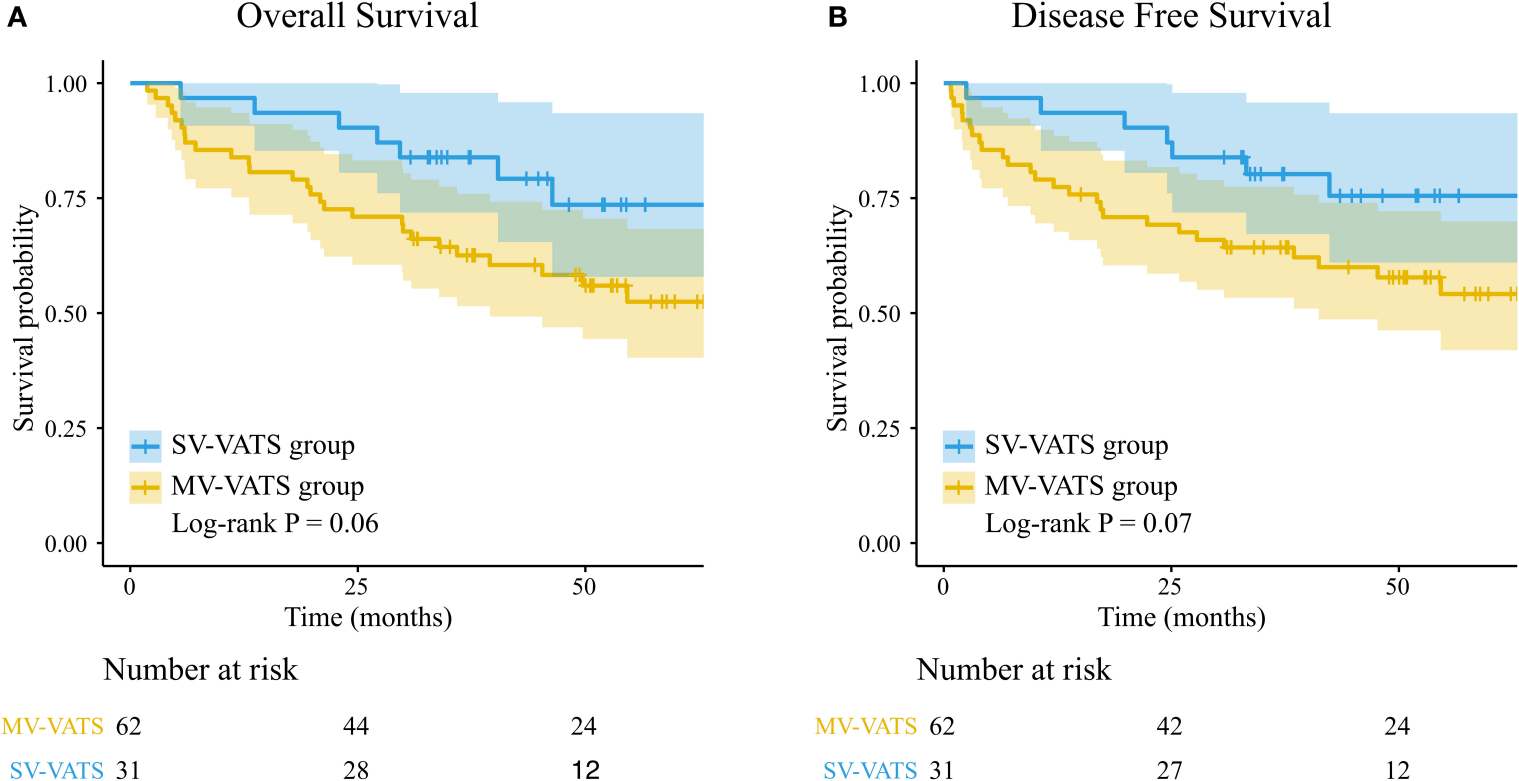
**(A)** K-M survival curves for overall survival in patients underwent the SV-VATS and MV-VATS. **(B)** K-M survival curves for disease-free survival underwent the SV-VATS and MV-VATS. K-M, Kaplan–Meier; SV-VATS, spontaneous ventilation video-assisted thoracoscopic surgery; MV-VATS, mechanical ventilation.

The median follow-up time was 46.83 (months) in the SV-VATS group and 50.97 (months) in the MV-VATS group. The median survival time and median disease-free survival time of patients underwent SV-VATS were 50.57 (months) and 50.25 (months), compared to 35.90 and 36.97 months in MV-VATS group. Kaplan-Meier analysis demonstrated no statistically significant difference between SV-VATS and MV-VATS groups in overall survival (OS) and disease-free survival (DFS). The 5-year OS and DFS curves were illustrated in Figure 2. Five-year survival analysis in the subgroups classified by FEV1 (%predicted) was shown in Supplementary Figure S3.

Univariate Cox regression revealed that OS (HR 0.46, 95% CI: 0.20–1.05, *P* = 0.07) and DFS (HR 0.46, 95% CI: 0.20–1.07, *P* = 0.07) were not statistically different between SV-VATS and MV-VATS groups. The results of multivariate analysis were: OS: HR 0.40, 95% CI: 0.17–0.95, *P* = 0.04; DFS: HR 0.78, 95% CI: 0.16 −0.90, *P* = 0.03.

## Discussion

This retrospective study compared the short- and long-term outcomes of NSCLC patients with poor lung function treated with SV-VATS and MV-VATS. The results indicated that SV-VATS generally exhibits comparable short-term effects, treatment prognosis, and long-term outcomes to MV-VATS, supporting its use in NSCLC patients with poor lung function.

VATS is a minimally invasive thoracic surgical technique that results in significantly less postoperative pain than conventional thoracotomy. Early outcomes such as postoperative and post-procedure infection can be improved by avoiding the rib spreader, muscle tissue division, and severing of intercostal (33, 34). In the traditional cognition, patients with poor lung function are unbearable to one-lung ventilation, not to mention VATS (35). Beyond this, other contraindications include morbid obesity, hemodynamic instability, extensive pleural adhesions, inability to cooperate, large centrally located tumors, a difficult airway, and others.

As the previous studies in our center on other thoracic patients undergoing SV-VATS, we concluded that non-intubated anesthesia VATS can shorten surgical operation and anesthesia time, reduce bleeding and optimize perioperative rehabilitation (12, 13, 18–20, 36). Studies from other centers also revealed that SV-VATS was associated with a shorter surgical time (37–39), and that it was a well-tolerated, feasible, and safe treatment option (39, 40).

However, few studies have been conducted to determine the feasibility and safety of SV-VATS in treating NSCLC patients with poor lung function. The applicability of SV-VATS in such patients remains uncertain. Wang et al. (41) demonstrated that non-intubated VATS for patients with impaired lung function was technically feasible, with a sample size of only 28 patients and without control group. As a consequence, further evidence is required to fully understand the feasibility and safety of SV-VATS in patients with poor lung function.

Our study demonstrated that SV-VATS could be feasible and safe for NSCLC patients with poor lung function according to intraoperative and postoperative short- and long-term outcomes.

Anesthesia is a key area that should be considered. The differences in anesthesia between the two groups are as follows: (i) No use of relaxant in the SV-VATS group. (ii) Reduced use of paroxysms in the SV-VATS group. The difference between the two types of anesthesia cannot be considered a confounding factor in the patient's postoperative outcome, as this study systematically compares the impact of the entire surgical procedure on patients, including different anesthesia types.

Patients undergoing SV-VATS had shorter anesthesia time and faster recovery, possibly due to its simplified preoperative preparation, such as no muscle relaxants, no bronchoscopy, and no endotracheal tube. Apart from this, the SV-VATS group received a decreased dose of major amnesic agents (propofol or sevoflurane) and regional anesthesia such as paravertebral without muscle relaxants (42). With a shorter anesthesia time, the SV-VATS group exhibits a less total amount of sedative medication, reducing side effects by avoiding over-dosage. Considered to be related to the prognosis of NSCLC patients, we collected their blood routine records to calculate SII, NLR, and PLR. The SV-VATS group had significantly lower NLR and PLR after surgery than that in the MV-VATS group in this study.

Only five patient undergoing SV-VATS experienced complications after surgery. No patient developed postoperative nausea and vomiting postoperatively, which may be related to the fact that all patients did not use morphine-based analgesics postoperatively (43, 44).

Patients undergoing SV-VATS have a shorter chest tube duration because of avoiding the intubation during the surgery, which can help to reduce the inflammatory responses and stress response (45, 46). Numerous types of thoracic procedures have demonstrated the advantages of SV-VAT (11). First, it could effectively reduce the intubation-related complications, such as atelectasis and ventilation-induced lung injury (47). Second, without using muscle relaxants (such as cis-atracurium), residual effects, including weakness of upper airway muscles and diaphragmatic, could be avoided to some extent (7, 48). Likewise, the advent of sugammadex may prevent the practice and prolonged paralysis risks. Third, SV-VATS provided better recovery for digestive and respiratory function due to the reduced use of major amnesic agents and muscle relaxants (49). Fourth, post thoracic surgery pain could be relieved for patients undergoing SV-VATS, possibly due to a better perioperative pain control by the paravertebral block (50).

Although SV-VATS is an emerging surgical technique, the surgical team should select the best surgical anesthesia method in the best interest of patients before performing SV-VATS. Besides, surgeons and anesthesiologists of the operation team should introduce the advantages and disadvantages of these two anesthesia methods to patients and their families according to the situation of patients before operation. The selection of surgical anesthesia method should be based on the willingness of the patients and their family members under the guidance of surgeon. In addition, several limitations of SV-VATS should be considered. First, chronic spontaneous breathing can result in hypercapnia (defined as PaCO_2_ ≥60 mmHg) and hypoxia (defined as SpO_2_ <90%). Second, since an uncontrollable ventilation situation is possible, conversion to general anesthesia with intubation may occur. Previous research indicated that the conversion rate was about 0–10% (51). Third, aspiration may occur without proper protection of the endotracheal tube, such as inadequate fasting, hiatal hernia, and gastroesophageal reflux disease.

We acknowledge several limitations in this study. First, this is a retrospective study. Although we used PSM, selection bias may exist. Second, as this is a retrospective study, we are unable to present some of the important outcome indicators, such as the patient's postoperative lung function and further indicators of lung function assessment, patient's postoperative pain, anesthesia, patient reported outcomes (PROs) or quality of recovery (QoR), data on intraoperative blood gases regarding ventilation/PaCO_2_ and acid/base which we will focus on in further studies. Third, since this is a single-center analysis, external validation should be established. Two randomized, multicenter trials are currently underway across 11 centers in Asia, focusing on NSCLC and lung bullae (NCT03432637 and NCT01677442), including our center. Third, a larger SD was observed in SII, NLR and PLR, which might affect our results to some extent. For that, blood routine data were double-checked to exclude the documentation error. The recent preoperatory and postoperative blood routine data for each patient were collected to observe the changes. However, different intervals between blood routine tests exist, implying a large SD.

## Conclusion

Our findings indicated that the SV-VATS is feasible and safe for NSCLC patients with poor lung function. Comparable short- and long-term outcomes implied that SV-VATS might be a viable alternative to MV-VATS when managing NSCLC patients with poor lung function.

## Central Message

We found that SV-VATS was comparable to MV-VATS in NSCLC patients with poor lung function, implying that SV-VATS might be a feasible alternative to MV-VATS for NSCLC patients with poor lung function.

## Perspective Statement

Poor lung function is a contraindication of SV-VATS. SV-VATS in such patients is controversial. We compared the short- and long-term postoperative outcomes between SV-VATS and MV-VATS in NSCLC patients with poor lung function. The results indicated the safety of SV-VATS in patients with poor lung function, implying a more extensive application of SV-VATS in such populations in the foreseeable future.

## Data Availability Statement

The datasets presented in this article are not readily available because; The patients involved in this study are from the Department of Thoracic Surgery of our hospital and are not open to anyone other than the author of this study. Requests to access the datasets should be directed to Runchen Wang, runchen_wang@outlook.com.

## Ethics Statement

The studies involving human participants were reviewed and approved by the Institutional Review Board of the First Affiliated Hospital of Guangzhou Medical University. Written informed consent to participate in this study was provided by the participants' legal guardian/next of kin.

## Author Contributions

RW: conceptualization, formal analysis, visualization, and project administration. QW and RW: writing–original draf and investigation. SJ: methodology and resources. CC: formal analysis. JZ: visualization. HLiu, JZ, CC, QW, RW, HW, and ZC: data curation. XL: methodology and software. HW: conceptualization. ZG: software. WL: validation. WL, WW, JH, and HLia: supervision and funding acquisition. WW, QW, RW, JZ, and HLia: writing–review. HLia, QW, RW, and JZ: editing. WW, JH, and HLia: resources and project administration. All authors contributed to the article and approved the submitted version.

## Funding

This work was supported by the Standardized Clinical Treatment Cohort Study (2017YFC0907903), China National Science Foundation (81871893 and 81501996), and Key Project of Guangzhou Scientific Research Project (201804020030).

## Conflict of Interest

The authors declare that the research was conducted in the absence of any commercial or financial relationships that could be construed as a potential conflict of interest.

## Publisher's Note

All claims expressed in this article are solely those of the authors and do not necessarily represent those of their affiliated organizations, or those of the publisher, the editors and the reviewers. Any product that may be evaluated in this article, or claim that may be made by its manufacturer, is not guaranteed or endorsed by the publisher.

## Supplementary Material

Supplementary Figure S1 Distribution of propensity scores.

Supplementary Figure S2 Propensity score before and after PSM.

Supplementary Figure S3 **(A)** K-M survival curves for overall survival in patients underwent the SV-VATS and MV-VATS graded according to FEV1 (%predicted). **(B)** K-M survival curves for disease-free survival underwent the SV-VATS and MV-VATS graded according to FEV1 (%predicted). K-M, Kaplan–Meier; SV-VATS, spontaneous ventilation video-assisted thoracoscopic surgery; MV-VATS, mechanical ventilation.

## Abbreviations

NSCLC: Non-small cell lung cancer
MV-VATS: Mechanical ventilation video-assisted thoracoscopic surgery
SV-VATS: Spontaneous ventilation video-assisted thoracoscopic surgery
SpO2: Pulse oxygen saturation
BIS: Bispectral index
TCI: Target-controlled infusion
SIMV: Synchronized intermittent mandatory ventilation
SII: Systemic immune-inflammation index
PSM: Propensity score matching
CT: Computed tomography
OS: overall survival
DFS: disease-free survival.
