# Supplementary material for: Spontaneous Ventilation Video-Assisted Thoracoscopic Surgery for Non-small-cell Lung Cancer Patients With Poor Lung Function: Short- and Long-Term Outcomes
Source: Front Surg. 2022 Mar 2;9:800082. doi: 10.3389/fsurg.2022.800082 (PMC8928927; doi:10.3389/fsurg.2022.800082)
Supplement: Supplementary file 1 [file Table_1.docx]

|  | **Unmatched patients in the SV-VATS group (10)** |
| --- | --- |
| Age (years) |  |
| < 60 | 1 |
| 60 - 75 | 15 |
| > 75 | 8 |
| BMI |  |
| < 18.5 | 2 |
| 18.5 - 25 | 22 |
| > 25 | 0 |
| Smoking status |  |
| Never smoking | 11 |
| Quit smoking | 5 |
| Smoking | 8 |
| Gender |  |
| Male | 21 |
| Female | 3 |
| Hypertension | 0 |
| Diabetes | 0 |
| T stage |  |
| 1 | 16 |
| 2 | 5 |
| 3 | 2 |
| 4 | 1 |
| N stage |  |
| 0 | 22 |
| 1 | 1 |
| 2 | 1 |
| 3 | 0 |
| M stage |  |
| 0 | 0 |
| 1 | 1 |
| ASA status class |  |
| Ⅰ | 1 |
| Ⅱ | 22 |
| Ⅲ | 1 |
| Surgical technique |  |
| Segmentectomy | 7 |
| Lobectomy | 24 |

Supplementary Table 1: Demographic information of unmatched patients in the SV-VATS group after PSM

SV-VATS: spontaneous ventilation video-assisted thoracoscopic surgery; BMI: body mass index; T: tumor; N: node; M: metastasis; ASA: American Society of Anesthesiologists.
