# Supplementary figures and images for: Spontaneous Ventilation Video-Assisted Thoracoscopic Surgery for Non-small-cell Lung Cancer Patients With Poor Lung Function: Short- and Long-Term Outcomes
Source: Front Surg. 2022 Mar 2;9:800082. doi: 10.3389/fsurg.2022.800082 (PMC8928927; doi:10.3389/fsurg.2022.800082)

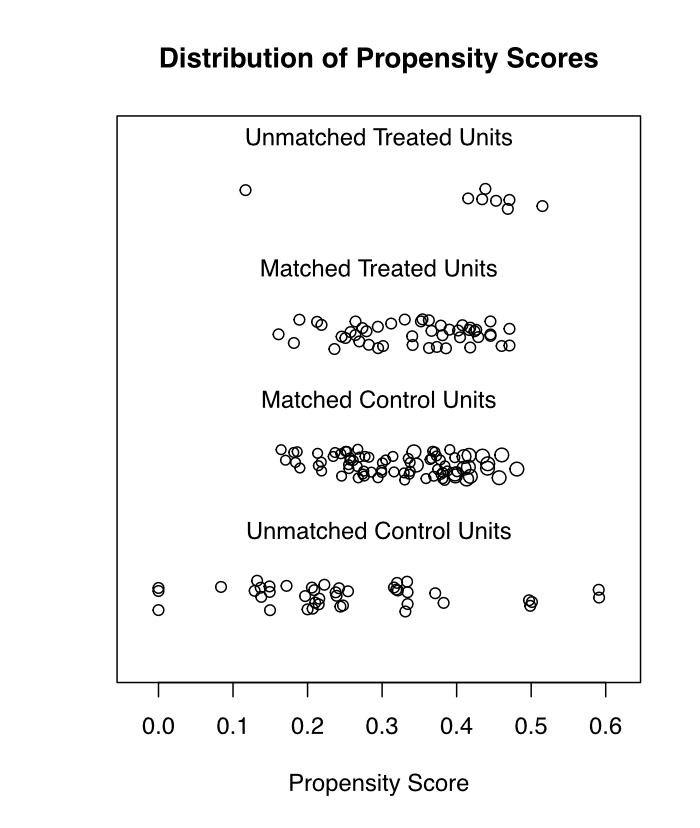

Supplement: Supplementary Figure S1 — Distribution of propensity scores. [file Image_1.TIFF]

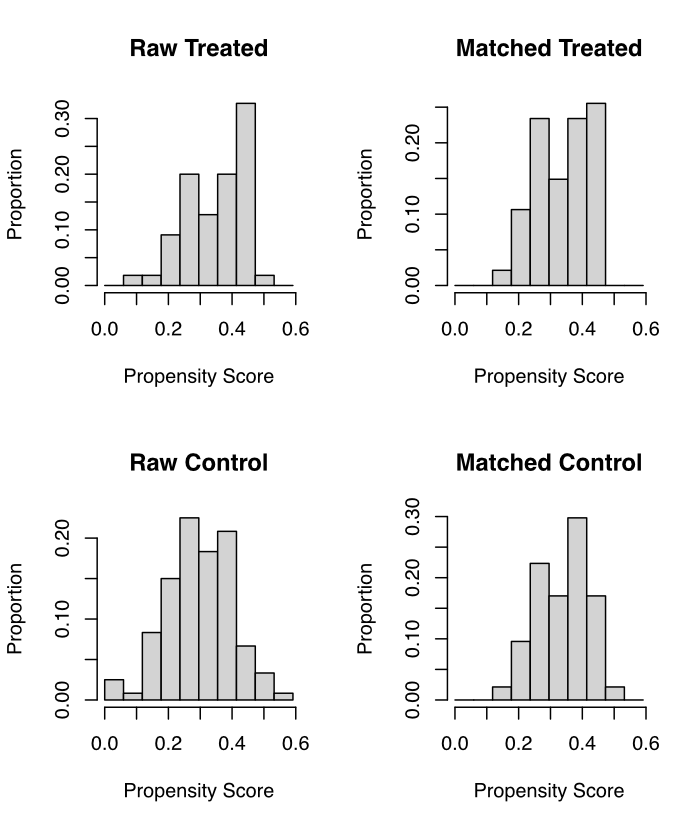

Supplement: Supplementary Figure S2 — Propensity score before and after PSM. [file Image_2.TIFF]

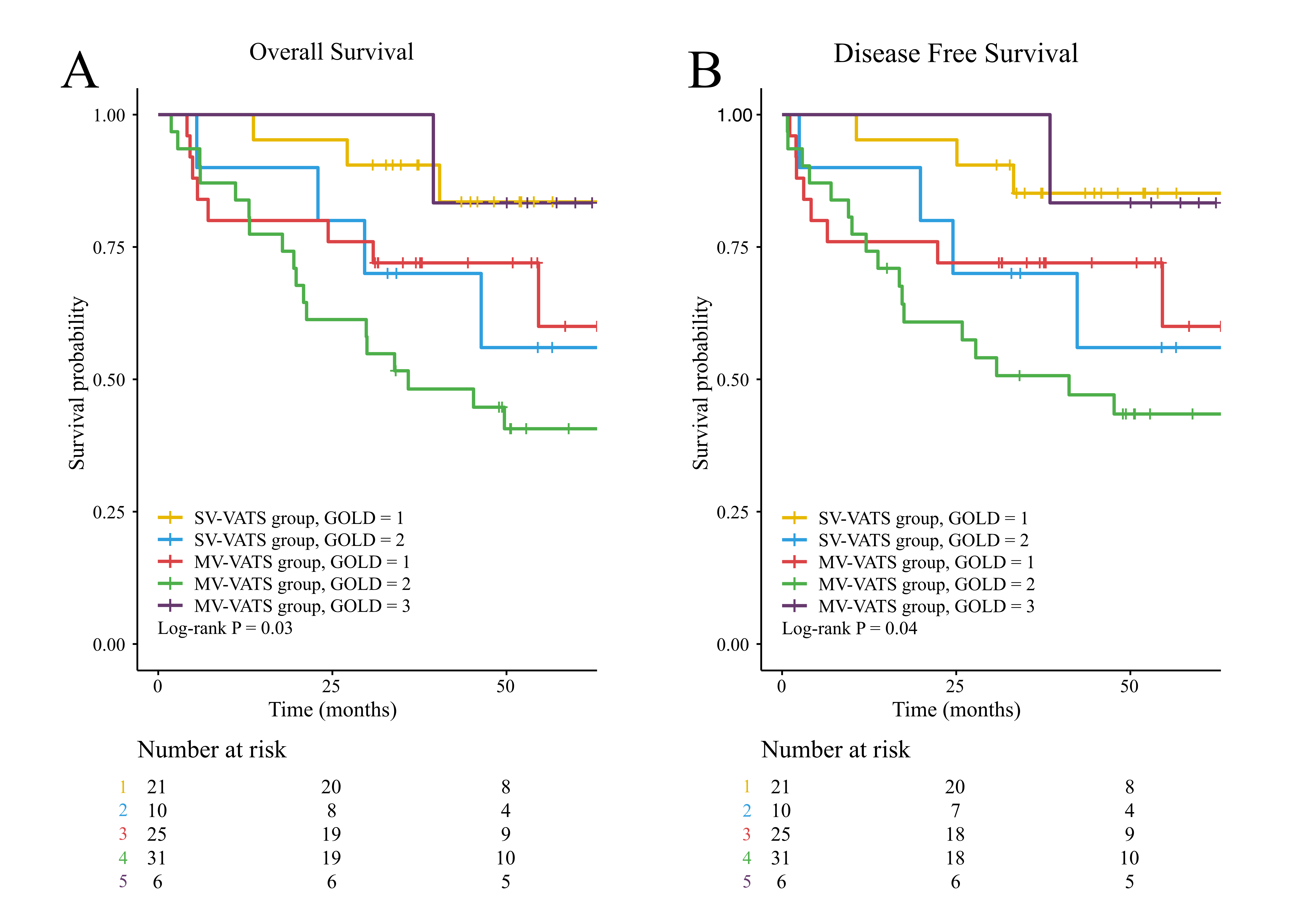

Supplement: Supplementary Figure S3 — (A) K-M survival curves for overall survival in patients underwent the SV-VATS and MV-VATS graded according to FEV1 (%predicted). (B) K-M survival curves for disease-free survival underwent the SV-VATS and MV-VATS graded according to FEV1 (%predicted). K-M, Kaplan–Meier; SV-VATS, spontaneous ventilation video-assisted thoracoscopic surgery; MV-VATS, mechanical ventilation. [file Image_3.TIFF]
